# New Hæmostatic Agent

**Published:** 1855-09

**Authors:** 


New Hæmostatic Agent.—M. Monsel, military pharmacien, (*Gaz. Med. de Paris, in Jour. de Chim. M.*) suggests the following solution as an efficient means of checking hæmorrhage:

℞ Tannic acid, ℈j,

Alum, (free from iron) ʒv,

Rose water, ℥xxv.

Dissolve. This solution also possesses a remarkable antiseptic power on the blood with which it comes in contact.

